# Essentials of the Principles and Practice of Medicine

**Published:** 1871-11

**Authors:** 


*Essentials of the Principals and Practice of Medicine.* A Hand-book for Students and Practitioners. By Henry Hartshorne, A. M., M. D. Third Edition, thoroughly revised. Philadelphia, Henry C. Lea, 1871.

The Third Edition of this work comes to us in a very neat, and pleasing form, comprising some four hundred pages of valuable and interesting information. In the preface to the Third Edition, Prof. Hartshorne says, "Great pains have been taken by the author, in prepairing the third edition of the "Essentials," to supply omissions, and to add whatever has seemed to be most positive and important in the recent advances of medical science. Such additions, however, although numerous, are conveyed in brief language, so as not to increase materially the size of the volume."

The general plan of the work is excellent, each subject being taken up and discussed in a liberal manner, although necessarily condensed in a work of this kind. We can heartily recommend it to both Students and Practitioners. It is a valuable companion to the student while attending lectures, and an aid to the young practitioner in the general treatment of disease.

